# Unraveling the Etiology of Pediatric Vertigo and Dizziness: A Tertiary Pediatric Center Experience

**DOI:** 10.3390/medicina57050475

**Published:** 2021-05-11

**Authors:** Nina Božanić Urbančič, Domen Vozel, Jure Urbančič, Saba Battelino

**Affiliations:** 1 Department of Otorhinolaryngology and Cervicofacial Surgery, University Medical Centre Ljubljana, Zaloška Cesta 2, SI-1000 Ljubljana, Slovenia; nina.bozanic@kclj.si (N.B.U.); domen.vozel@kclj.si (D.V.); jure.urbancic@kclj.si (J.U.); 2 Faculty of Medicine, University of Ljubljana, Vrazov Trg 2, SI-1000 Ljubljana, Slovenia

**Keywords:** dizziness, vertigo, migraine disorders, interdisciplinary communication, headache, medulloblastoma, Lyme neuroborreliosis, somatoform disorders, child, adolescent

## Abstract

*Background and Objectives*: Numerous authors have reported that the commonest type of vertigo in children is migraine-associated vertigo (vestibular migraine and benign paroxysmal vertigo of childhood—BPV). We aimed to provide the possible etiological background of vertigo and dizziness in Slovenian children. *Materials and Methods*: A retrospective case series of pediatric vertigo and dizziness children referred to the tertiary pediatric otorhinolaryngology center from 2015 to 2020. Children received a complete audiological and vestibular workup and were referred to pediatric specialists depending on the clinical presentation. *Results*: Of 257 children (42% male, 58% female) aged 1–17 years (M = 10.9, SD = 4.3 years) in 19.1% vertigo and dizziness were classified as central, in 12.4% as a peripheral vestibular, in 10.9% as a hemodynamic, in 5.8% as a psychological and none as visual by pediatric neurologists, otorhinolaryngologists, cardiologists, psychologists or ophthalmologists, respectively. 40.8% (20) children with central vertigo had BPV (7.8% of all children) and 8.2% (4) migrainous vertigo. In 43.6% (112 children), the etiology remained unclassified. *Conclusions*: After a thorough multidisciplinary workup, the etiology of vertigo and dizziness was unraveled in the majority of children referred to our tertiary otorhinolaryngology center. The most common cause was central; however, in a considerable number, the etiology remained unclassified. The latter could be attributed to the self-limiting nature of vertigo spells. Hence, a child presenting with dizziness and vertigo requires a multidisciplinary approach, in which referral to a neurologist is, in most cases, essential.

## 1. Introduction

Good balance is crucial for a child’s normal development and psychophysical wellbeing. The symptoms of dizziness and vertigo are not as common in children as they are in the adult population. The prevalence of vertigo in children is said to be from 5 to 15% [1,2]. As has been proven by an American National Health Interview Survey, children with vertigo have significantly higher chances of attention deficit disorder, learning problems, developmental delay, and intellectual disability. They also have higher odds of having difficulty with emotions, concentration, or behavior and having a poor attention span [3]. As some experiments have shown, central vertigo is also critical in relation to numerical skills [4].

Due to the identified significant impact of balance disorders, vertigo, and dizziness on the child’s psychophysical health, the management of this disease necessitates a multidisciplinary approach. This approach encompasses pediatric, neurological, otorhinolaryngological, psychological, cardiological, and ophthalmological evaluation to unravel the etiology and establish the diagnosis of vertigo.

The causes of vertigo and dizziness in children are diverse and require attention from various specialists. As reported by numerous authors, the commonest type of vertigo in children is said to be migraine-related vertigo (vestibular migraine and benign paroxysmal vertigo of childhood—BPV) [2,5,6,7,8,9,10,11,12,13,14]. Vestibular migraine presents with vestibular symptoms, occasionally associated with migrainous headache [14,15].

In BPV, a two to six-year-old child has short vertigo spells (seconds to minutes), often accompanied by nystagmus and imbalance. It is frequently associated with a positive family history of migraine headaches and the later development of typical migraine [16]. 

Other central causes of vertigo and dizziness in children include epileptic, infectious (including Lyme borreliosis), neoplastic, vascular (e.g., malformation), postoperative vertigo, vertigo due to hydrocephalus, degenerative/hereditary vertigo, and several others [5,17,18].

Vertigo and dizziness can present as epileptic aura, usually in children with partial seizures, the temporal lobe being the most frequent ictal onset area [19]. They can also be a consequence of post-traumatic, autoimmune, hormonal, pharmacological, and numerous other pathologies [3,15,17,18,20,21].

Lyme neuroborreliosis is a tick-borne infection of the nervous system caused by *Borrelia burgdorferi* spirochetes. Vertigo as a sign of Lyme borreliosis is more common in adults but can also appear in children [22]. It should not be forgotten in the differential diagnosis of pediatric vertigo. 

Vestibular symptoms can arise because of lesions in the brainstem and the cerebellum, the medulloblastoma being one of the most common tumors of childhood. Head imaging is a necessary part of diagnostic workup when such a lesion is suspected [5].

Frequent vertigo episodes (up to 30 per day), lasting for seconds to minutes, are rare and can be caused by vestibular nerve vascular compression—a syndrome termed vestibular paroxysmia [5,6].

The most common cause of hemodynamically caused dizziness is orthostatic hypotension, responsible for 3 to 9% of symptomatic children 12 and can occur even in children without autonomic dysfunction [23]. 

The other common cause of vertigo and dizziness in children, as well as in adults, is psychological problems [5]. On the other hand, vertigo (of different origins) can lead to psychological distress - symptoms of depression and anxiety [24]. It is most prevalent among adolescent girls [9].

Peripheral vestibular causes of vertigo are uncommon in children, with vestibular neuronitis occurring only in 1–5% of pediatric vertigo cases [25]. 

Due to the abovementioned broad spectrum of pediatric vertigo and dizziness causes, the main aim of this retrospective study was to provide the possible etiological background of vertigo and dizziness in Slovenian children.

## 2. Materials and Methods

That is a retrospective study of consecutive pediatric vertigo and dizziness patients (aged 1–17 years) presenting at the tertiary pediatric otorhinolaryngology center (from January 2015 till November 2020).

The diagnostic workup started with detailed history taking. We were especially interested in problems concerning balance described by parents, who had sometimes even filmed a vertigo episode on a mobile phone. We also noted the child’s description of the symptoms since they can be quite picturesque and narrative. We were also interested in possible oncological therapies, head trauma, headaches, family history of migraines, and previous intracranial operations.

Each child presenting at our clinic received an otoneurological clinical examination, immittance testing by tympanometry (Interacoustics Impedance Audiometer AT235, Middelfart, Denmark), followed by audiovestibular testing tailored to their age and capacity to cooperate.

The otoneurological clinical examination consisted of otomicroscopy (OPMI pico Zeiss, 7743 Jena, Germany), detailed otorhinolaryngological examination, and related cranial nerve function and nystagmus assessment using video Frenzel goggles (Interacoustics, Middelfart, Denmark), Romberg’s test, and tandem walking with eyes open and closed. 

The function of the semicircular canals was tested using low-frequency stimulus (caloric testing, on Variotherm plus ATMOS MedizinTechnik GmbH and Co. KG Ludwig-Kegel, D-79853 Lenzkirch/Germany and rotatory on a rotatory chair) and high-frequency testing (video head impulse test on Interacustics, 5500 Middelfart, Denmark). The otolith organs were assessed by subjective visual vertical test (Virtual S.V.V. Interacustics A7S, 5500 Middelfart, Denmark) and cervical evoked myogenic potentials testing (cVEMP, Eclipse, Interacustics A/S, 6610 Assens, Denmark).

A peripheral vestibular disorder was diagnosed when at least one of the performed vestibular tests was abnormal. When a peripheral vestibular disorder was excluded, the child was referred to other subspecialists, i.e., a pediatric neurologist and, depending on the signs and symptoms, to ophthalmologic, infectious disease (serology results of Borrelia Burgdorferi), radiological (CT and MRI), psychiatric, psychological, and cardiological consultations, as part of a multidisciplinary approach.

A pediatric neurologist performed the neurological examination, followed by EEG, laboratory (serology to *Borrelia Burgdorferi*), and sometimes head imaging (CT/MRI).

For the diagnosis of pediatric migraine, we use adapted IHS (International Headache Society) criteria [26]. We also have our questionnaire for parents of children with headache/suspected migraine (in Slovenian language). Developmental quotient assessment was used according to gross motor abilities (motor age/chronological age × 100), e.g., an infant who was 12 months old and has just started to sit scored 50. Our psychologists used the Bayley II assessment at the age of 18 months [27].

The children were given a multidisciplinary follow up until the symptoms subsided, as necessary.

Statistical analysis was done using SPSS V20.0 (IBM, Armonk, NY, USA) and Microsoft Excel 2019 (Microsoft, Redmond, WA, USA). *p* < 0.05 was considered as a statistically significant difference.

## 3. Results

This retrospective study included 257 (42% male, 58% female) children aged between 1 and 17 years (M = 10.9, SD = 4.3 years) referred to our tertiary otorhinolaryngology center in 2015–2020 time period presenting with vertigo and dizziness. 

**Figure 1:**
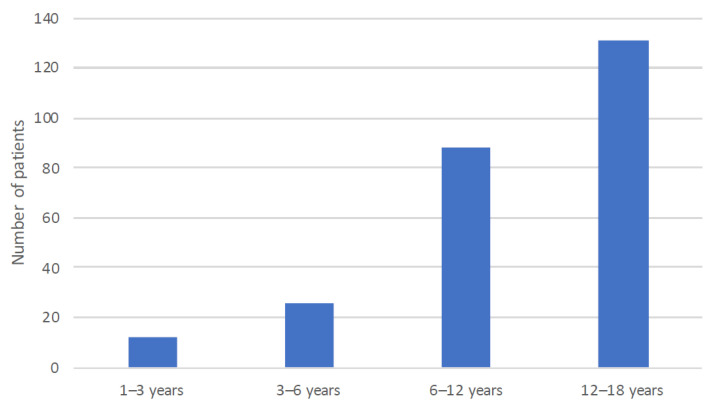
Age distribution of pediatric vertigo and dizziness.

The age distribution is depicted in Figure 1.

In 182 (70.8%) children, there was no nystagmus visible with video Frenzel goggles; in 40 (15.6%), there was no data, and in 35 (13.6%), the nystagmus was detected. In 17 (6.6%) children, the nystagmus had non-peripheral characteristics (not horizontal or not following Alexander’s law) and in 18 (7.0%) of peripheral type. A Romberg test was performed in 188 (73.2%) children and found pathological in 26 (13.8%) performed tests. The tandem walking test was performed in 178 (69.2%) and found pathological in 59 (33.1%) performed tests. 

The data about the headache was obtained in 165 (64.2%) children. 100 (60.6%) of these children had reported no headache, and 65 (39.4%) reported a headache. The headache was classified as a migraine in 55 (84.6%) and as other types in 10 (15.4%) children. 

The family history of migraine was obtained in 91 (35.4%) children; of these, 49 (53.8%) children had a positive family history. 

**Figure 2:**
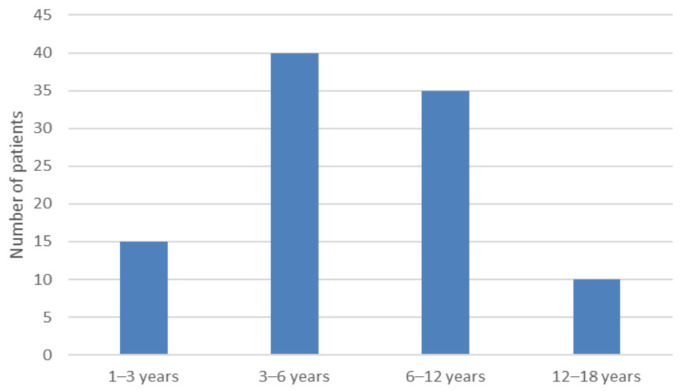
Age distribution of benign paroxysmal vertigo of childhood (BPV). None under 1-year-old was diagnosed as BPV.

Two (3.6%) children with migraine headaches were diagnosed with BPV, and 20 (40.8%) with a positive family history of migraine were diagnosed with BPV. Its age distribution is shown in Figure 2.

**Figure 3:**
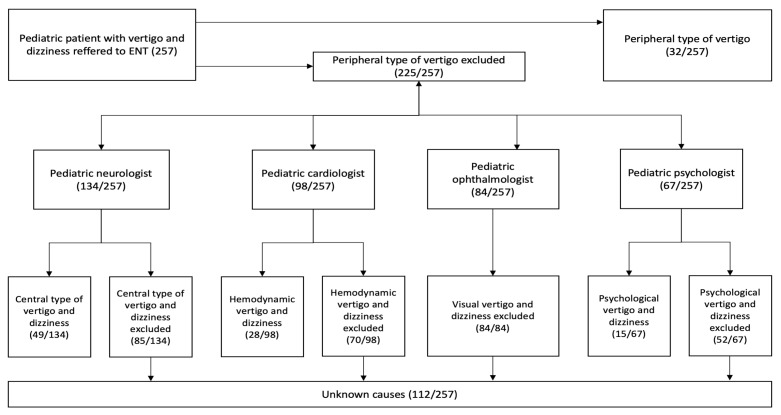
Flowchart showing the number of children that were referred through each of the sub-specialties.

A total of 32 (12.4%) children were diagnosed with peripheral vestibular vertigo. The number of children referred to each of the subspecialties is depicted on the flowchart (Figure 3). 

A total of 134 (52.1%) children were referred to a pediatric neurologist (Figure 3), wherein the neurological examination was flawless in 117 (87.3%) children. In 50 (37.3%) children, an EEG was done, which was pathological in 9 (18.0%). Only in one child, the dizziness and vertigo symptoms could be explained by the pathological EEG. Overall, 49 (19.0%) children were diagnosed with a central type of vertigo, which presented 36.6% of children referred to a pediatric neurologist. A total of 20 (40.8%) of children with a central type of vertigo had BPV, and 4 (8.2%) had a migrainous type. Overall, 7.8% of children had BPV.

A total of 98 (38.1%) children were referred to a cardiologist, 28 (28.5%) of them being diagnosed with hemodynamic dizziness, which presented 10.9% of all children in our study.

A total of 84 (32.6%) children were referred to an ophthalmologist, 12 (14.2%) of them being reported to have abnormal findings (i.e., astigmatism, anisometropia, amblyopia, blindness, trochlear nerve paresis, sicca syndrome, and papilledema). However, no ophthalmological abnormality was related to vertigo and dizziness. 

A total of 67 (26.1%) children were referred to a psychologist, 15 (22.4%) of them being diagnosed with psychogenic vertigo and dizziness, which presented 5.8% of all children included in our study.

In terms of head imaging, 109 (42.4%) had an MRI of the head performed, of which 18 (16.5%) children had some pathological finding (excluding cysts of the pineal gland). 47 (18.2%) children had a CT scan done, with only two (4.2%) of them being pathological. Overall, in 20 (7.7%) children, head imaging (CT or MRI) revealed a pathology.

A total of 17 (6.7%) had a head trauma preceding the onset of vertigo.

Nine (4%) children had had previous intracranial operations, and seven (3%) had positive serology to *Borrelia Burgdorferi*.

Regardless of the multidisciplinary workup, our children go through, in 43.6% of children, the etiology of vertigo and dizziness remained unknown.

**Figure 4:**
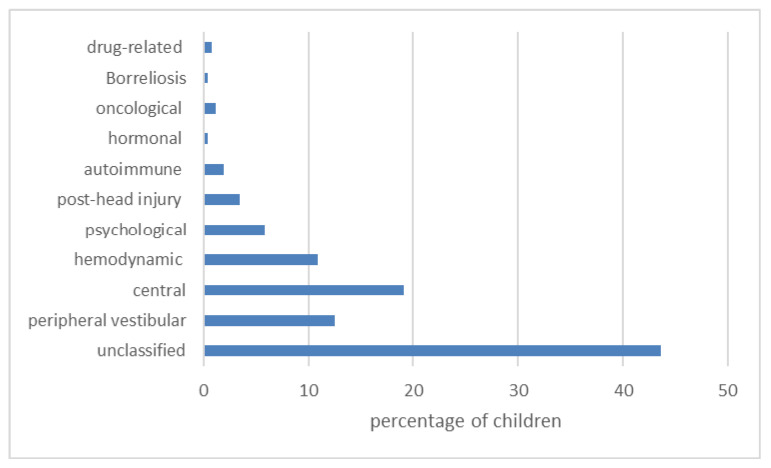
The etiology of pediatric vertigo and dizziness. Numbers represent the percentage of each etiology among 257 referred children.

**Figure 5:**
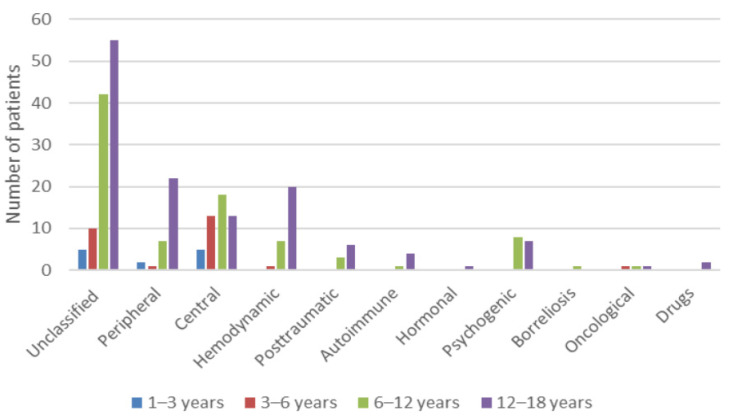
Age distribution stratified by the etiology of pediatric vertigo and dizziness.

Figure 4 shows the etiology of pediatric vertigo and dizziness, and in Figure 5, an additional age distribution stratified by the etiology.

**Figure 6:**
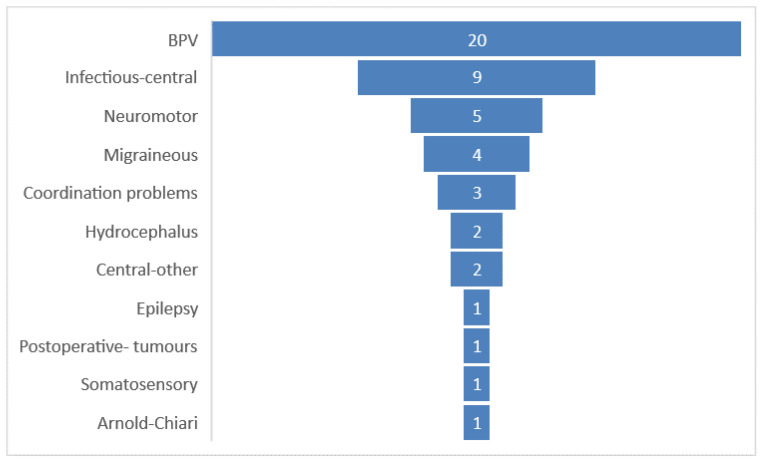
Different central pathologies found in our children. The graph shows the number of each central pathology of vertigo and dizziness.

**Figure 7:**
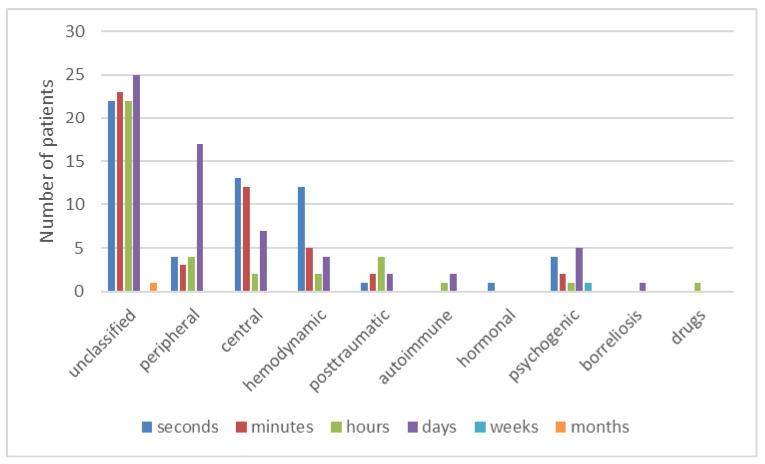
Duration of vertigo and dizziness symptoms stratified by its etiology.

There were several different pathologies involved in a definite diagnosis of central loss, as shown in Figure 6. Figure 7 shows the duration of vertigo and dizziness stratified by its etiology.

## 4. Discussion

This retrospective study provides the background of pediatric vertigo and dizziness etiology. The latter was unraveled in the majority of children referred to our tertiary otorhinolaryngology referral center. The most common cause was central. However, in a considerable number of children, the etiology of vertigo and dizziness remained unclassified. 

The central pathology, unraveled in 19% of patients, was lower than in some studies [18]. The most common type of central vertigo was BPV, as in numerous other studies [2,6,11,13,14]. In general, headaches are often encountered in children and adolescents, as shown by Lee et al. [24]. It also one of the major symptoms in a subset of patients with pediatric vertigo and dizziness, comprising 35–60% of cases [1]. Our patients had occasional headaches in 39.4%, and 84.6% of them were migrainous. Overall, 21.4% of all children had a migrainous headache, comparable to the literature [11]. Children only rarely have a classic migraine [25], which was diagnosed in 1.6% of our group. 

Migraine-related syndromes as BPV have also been described as the most prevalent cause of vertigo and dizziness in children aged 2–6 [6], also substantiated by our study [25]. The etiology of BPV are presumably vascular alterations resulting in transient hypoxia of vestibular pathways and nuclei [28] or by ischemia of utricular otoconia [2]. A total of 7.8% of our pediatric patients had a final diagnosis of BPV, which is lower than reported elsewhere [29].

We had only one child with balance problems due to Arnold–Chiari malformation (ACM), which is much less than in other studies. It accounted for disequilibrium and vertigo in 10% of patients [12]. In general, motor, sensory and developmental problems account for 11% of vertigo and dizziness, according to published reports [17]. Similar to the previously mentioned ACM, we had only a minority, nine children (3.5%), with these types of disorders (i.e., neuromotor, somatosensory, and coordination problems). Impaired cerebrospinal fluid circulation, including hydrocephalus, also seems to play a minor role in our group of patients (2/257) [30]. In pediatric vertigo cases with an alteration of consciousness, an EEG should be done to rule out epilepsy. In our rather large group of patients, we found only one child with vertigo due to epilepsy. In some other studies, an epileptic pathology was more frequent [7].

Head imaging is frequently used to evaluate children with vertigo and dizziness. However, the etiological background can be confirmed rarely. Our study revealed the relevant pathology in rare cases (20 patients, 7.7%), which is comparable to some other studies [25]. Only one child in our group of patients had vertigo and dizziness symptoms due to neoplastic disease (i.e., medulloblastoma). Given the need for sedation in young children and CT’s ionizing radiation, these diagnostic procedures should only be directed towards selected cases. 

Interestingly, vertigo symptoms usually subside spontaneously in some children [29], which in our opinion also contributed to the unclassified etiology in 43.6% of children. Interestingly, other authors have reported unclassified etiology in 13–18% [10,31]. Otoneurological clinical assessment for a peripheral vestibular disorder was negative in these children. Additionally, they did not have any signs of the neurological deficit during the neurological workup. In the patient’s history, there was nothing to support the diagnosis of orthostatic or psychological involvement. One possible explanation of this discordant result is that our study did not enroll children with acute otitis media, its complications, and acute head trauma (e.g., temporal bone fracture), which can all cause about 15% of dizziness and vertigo [31,32]. Figure 7 shows the short duration of symptoms in the »unclassified« group of patients. In our group of patients, vertigo and dizziness were more common in older children (12–18 years). In these children, vertigo and dizziness remained unclassified in the majority. We believe that they probably had minor brief transient psychological or hemodynamic (orthostatic) problems, which could be underdiagnosed in pediatric vertigo and dizziness [6,33]. All the children with the symptoms of vasovagal-orthostatic dizziness were given instructions about alleviating the symptoms. Moreover, it is clear from Figure 5 that both psychological or hemodynamic vertigo appears primarily in the teenage group, the same as unclassified vertigo.

Retrospective study design and involvement of multiple specialists in comprehensive workup may reflect in loss of potentially critical data, which is the main limitation of our study. It would be desirable to make a prospective multicentric study with a data collection registry to make the research more robust in the future.

The strengths of our study are the group size, which was large for Slovenia’s population of 2 million people and large compared to other studies [7,9,10,18,29,31]. Another strength is a multidisciplinary workup provided to pediatric vertigo and dizziness patients in our institution.

## 5. Conclusions

Balance is crucial for a child’s normal psychophysical development. Due to the numerous possible causes, a child presenting with dizziness and vertigo requires a multidisciplinary approach, in which referral to a neurologist is essential. Peripheral vestibular etiology is rare and central etiology is the most common. In many children, especially teenagers, pediatric vertigo and dizziness remain unclassified. This could be attributed to underdiagnosed hemodynamic or psychological etiology or simply the self-limiting nature of vertigo and dizziness in children. 

## Data Availability

The data presented in this study are available on request from the corresponding author.
